# A Rodent Model of Dynamic Facial Reanimation Using Functional Electrical Stimulation

**DOI:** 10.3389/fnins.2017.00193

**Published:** 2017-04-05

**Authors:** Mark A. Attiah, Julius de Vries, Andrew G. Richardson, Timothy H. Lucas

**Affiliations:** Department of Neurosurgery, Center for Neuroengineering and Therapeutics, Perelman School of Medicine, University of PennsylvaniaPhiladelphia, PA, USA

**Keywords:** paralysis, facial nerve, functional electrical stimulation, electromyography, rodent

## Abstract

Facial paralysis can be a devastating condition, causing disfiguring facial droop, slurred speech, eye dryness, scarring and blindness. This study investigated the utility of closed-loop functional electric stimulation (FES) for reanimating paralyzed facial muscles in a quantitative rodent model. The right buccal and marginal mandibular branches of the rat facial nerve were transected for selective, unilateral paralysis of whisker muscles. Microwire electrodes were implanted bilaterally into the facial musculature for FES and electromyographic (EMG) recording. With the rats awake and head-fixed, whisker trajectories were tracked bilaterally with optical micrometers. First, the relationship between EMG and volitional whisker movement was quantified on the intact side of the face. Second, the effect of FES on whisker trajectories was quantified on the paralyzed side. Third, closed-loop experiments were performed in which the EMG signal on the intact side triggered FES on the paralyzed side to restore symmetric whisking. The results demonstrate a novel *in vivo* platform for developing control strategies for neuromuscular facial prostheses.

## Introduction

Facial paralysis is a disfiguring condition affecting 127,000 individuals annually (Bleicher et al., 1996). Beyond cosmetic disfigurement, facial paralysis can lead to permanent disability. Blindness may occur if the cascade of corneal dryness, conjunctivitis, and ulceration develops (Otto et al., 1986). Nasal obstruction and mouth leakage result from an inability to keep nasal passages patent and mouth closed, respectively (May et al., 1977). Finally, dysarthria and distorted facial expressions hinder both spoken and nonverbal communication (Coulson et al., 2004), resulting in awkward and strained social interactions.

The ultimate goal of facial reanimation is to enable independent control of paralyzed facial muscles. This goal has proven elusive. Development of effective treatment options is confounded by the wide range of causes that result in a shared facial palsy phenotype, namely, trauma, infection, neoplasm, iatrogenic insults and idiopathic etiologies (Melvin and Limb, 2008). Current treatments palliate the condition by partially restoring muscle tone or by attempting to prevent catastrophic consequences, like blindness. These procedures all have significant drawbacks. Sacrifice of the hypoglossal nerve for nerve transfer results in ipsilateral tongue paralysis. Cross-facial nerve grafts route contralateral facial branches to the paralyzed side, but this procedure places the healthy nerves at risk (Hontanilla et al., 2013). Dynamic procedures routinely take 9–18 months to become effective and require rigorous rehabilitation (Spector et al., 1991; Robey and Snyder, 2011). Spontaneous movement or emotional expression is rarely achieved and the functional status is often compromised by synkinesis. To improve outcomes researchers are developing several new treatments (Hadlock and Cheney, 2008), including the use of functional electrical stimulation (FES) to reanimate paralyzed facial muscles (Griffin and Kim, 2011).

The effectiveness of FES in activating paralyzed facial muscles, in particular the orbicularis oculi to restore eye blink, has been demonstrated in rabbits and dogs (Rothstein and Berlinger, 1986; Salerno et al., 1991; Otto, 1997; Somia et al., 2001; Sachs et al., 2007; Jie et al., 2016). Since many facial movements including eye blink are symmetric, a natural closed-loop FES control signal in unilateral facial paralysis can be derived from the intact side of the face. In particular, electromyographic (EMG) recordings from a facial muscle contralateral to the injury can be used to trigger FES of the homologous paralyzed muscle. A similar, bilateral, closed-loop approach has been used to rehabilitate hand movements after stroke (Knutson et al., 2012, 2016). In the context of facial paralysis, this closed-loop strategy has been demonstrated in rabbits, dogs, and humans (Zealear and Dedo, 1977; Tobey and Sutton, 1978; Broniatowski et al., 1987, 1989, 1991; Cao et al., 2009; McDonnall et al., 2009; Kurita et al., 2010; Frigerio and Cavallari, 2012; Yi et al., 2013). Most of these studies focused on restoration of eye blink and utilized largely qualitative measures of FES performance.

In the present work, we sought to develop a new, quantitative animal model of FES-controlled facial reanimation. Quantitative tracking of bilateral facial movements is typically done with video cameras and feature recognition software (Sachs et al., 2007). But this strategy is error-prone and has low temporal resolution. An alternative strategy is to track a more conspicuous facial feature present on some animals: facial vibrissae (whiskers). Rodent whiskers are controlled by muscles innervated by the facial nerve (Berg and Kleinfeld, 2003), typically exhibit bilateral symmetric motion (Gao et al., 2001), and can be readily tracked with high spatiotemporal resolution (Bermejo et al., 1998). Thus, we hypothesized that a rodent model of unilateral facial paralysis could provide an improved assessment of FES performance for dynamic facial reanimation. A similar approach with rats was developed previously for the study of facial nerve function (Heaton et al., 2008). In the present study we quantified, for the first time, the effects of open- and closed-loop FES on whisker motion. The results suggest this new approach could be useful for designing improved FES control policies for a facial prosthesis.

## Methods

All procedures were approved by the University of Pennsylvania Institutional Animal Care and Use Committee. Four female Sprague-Dawley rats were used for these experiments.

### Training and experimental apparatus

**Figure 1:**
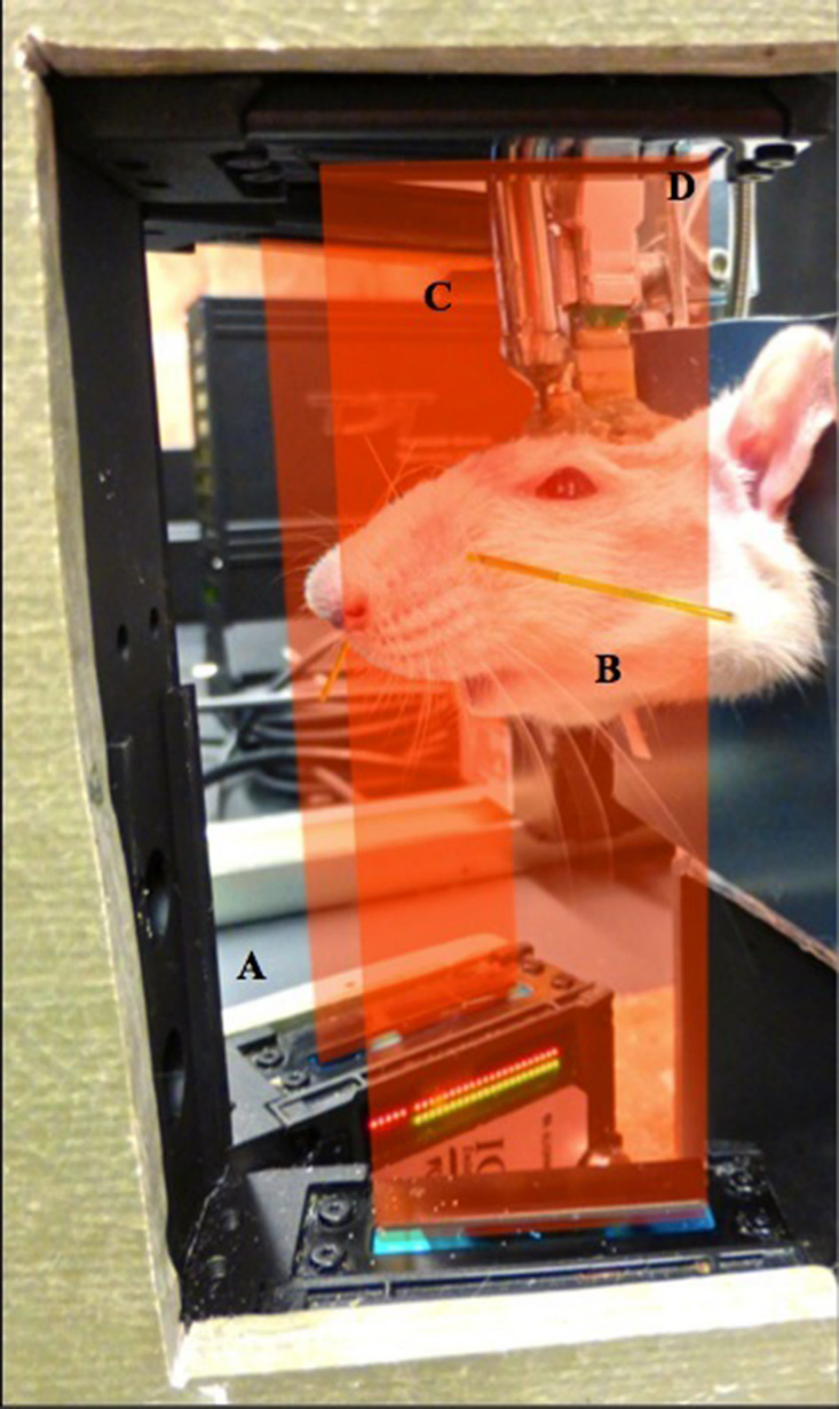
**Experimental setup. (A)** Laser micrometers positioned on either side of the face were used to monitor whisker movement. **(B)** Polyimide tubes were placed on selected whiskers and micrometer sensitivity was adjusted such that only the motion of one pair of whiskers was detected. **(C)** Head restraint was achieved using magnetic implant and a repositionable rod. **(D)** Microwire electrodes implanted in facial muscles were accessed via a connector anchored to the skull.

Prior to surgery, all animals were habituated to the laboratory environment and the experimental apparatus. The apparatus (Figure 1) consisted of (1) a half-pipe with hook-and-loop strap to restrain the rat's body, (2) neck plates at the front of the pipe to restrain forward or backward head motion, (3) a metal rod with magnet that mated with a head-mounted magnet to further restrain the head, and (4) two commercial laser micrometers in a V-configuration such that they approximated both sides of the face (see details below). The half pipe could be moved to adjust the position of the rat relative to the micrometers. A Faraday cage, which enclosed the entire apparatus, was assembled with copper mesh and red translucent acrylic in order to shield recordings from ambient electromagnetic interference and to provide an enclosed environment that minimized the stress of the animals. Rats were placed in the restraints each day until they were able to be calm for at least 1 h.

### Implant

For each rat, a custom microwire array was constructed to interface with the facial musculature. Seventeen insulated stainless steel wires (50 μm, A-M Systems) were soldered to a printed circuit board (PCB, 6.68 × 7.05 × 1.59 mm) and routed to a miniature connector on the board (A79042-001, Omnetics Corp). Sixteen of the wires served as working electrodes in facial muscles (eight on each side) and one as a reference electrode. An uninsulated silver wire (127 μm, A-M Systems) was also soldered to the PCB to serve as the ground. Wires were impedance tested to ensure the integrity of the electrical connections. The PCB was covered with an epoxy to insulate and stabilize the solder connections and traces. The distal 2 mm of the stainless steel wires was stripped of insulation. Before surgery the implant was cold sterilized in chlorhexidine (0.1%).

### Surgical procedure

Our development of the rodent model proceeded in two sequential phases: (1) establish the techniques for bilateral recording of facial EMG and whisking motion (animals 1 and 2) and (2) paralyze and implement open- and closed-loop FES (animals 3 and 4). As such, the facial nerve transection portion of the surgical procedure applied only to animals 3 and 4.

Each rat was anesthetized with intraperitoneal administration of ketamine (60 mg/kg) and dexmedetomidine (0.25 mg/kg). The animal was kept on a heating pad and the respiratory rate, palpebral reflex, and pedal pinch reflex were monitored periodically throughout the procedure to track depth of anesthesia. Once a surgical plane of anesthesia was reached, the head and right side of the face posterior to the whisker pad were shaved and cleaned with povidone-iodine. Lidocaine was administered subcutaneously at the incision sites for further analgesia.

The buccal and marginal mandibular branches of the facial nerve, the only branches supplying motor input to the whisker pad (Semba and Egger, 1986), were exposed through a single vertical skin incision on the right side of the face. Nerve identity was confirmed with bipolar stimulation, which elicited movement from the whisker pad. A 3 mm section of each nerve was transected. Functional denervation was confirmed again with bipolar stimulation to the proximal and distal stumps. The incision was closed with 6-0 nylon suture.

The animal was then placed in a stereotaxic frame. An incision was made on the midline of the scalp and skin, soft tissue, and periosteum were retracted laterally to expose the frontal and parietal bones. Six burr holes were made on the skull bilaterally and six screws (00-90 × 1/8″) were inserted. The implant PCB was positioned in the middle of the skull screws and the silver ground wire was wrapped around the screws. To aid head fixation, a high-strength cylindrical neodymium magnet (D34-N52, K&J Magnetics Inc.) was placed rostral to the PCB. Dental acrylic was then poured over the PCB, screws, and around the magnet to secure them to the skull.

The stainless steel microwires were then inserted into the muscles of the whisker pad in pairs. The hooked ends of each pair of wires were placed in a 23-gauge hypodermic needle that was used to drive them into the targeted muscle (Carvell et al., 1991). The targets initially included both intrinsic and extrinsic muscles of the whisker pad, which protract and retract the whiskers, respectively (Dörfl, 1982). However, in preliminary studies we found that actively controlling the retractor muscles, nasolabialis and maxillolabialis, introduced significant complexity (see Section Discussion). Whisker retractions can occur passively, due to the viscoelastic properties of the facial connective tissue (Bermejo et al., 1998). Accordingly, the intrinsic muscles were targeted preferentially. Intramuscular placement was confirmed with bipolar stimulation that evoked whisker protraction. The stainless steel reference wire was placed subcutaneously on the dorsal aspect of the nasal bone. The midline incision was closed with 6-0 nylon sutures.

Anesthesia was reversed with atipamezole (5 mg/kg). For 3 days postoperatively, animals were maintained on an analgesic regimen of ketoprofen (5 mg/kg, once daily) and buprenorphine (0.05 mg/kg, twice daily), administered subcutaneously. To prevent infection, gentamicin sulfate drops (0.3%, once daily) were applied around the implant.

### Whisker monitoring

After surgery, the rats were further acclimated to the experimental apparatus via training sessions in which their heads were held stationary by the magnetic attachment (Figure 1). Horizontal whisker motion was monitored bilaterally by two laser micrometers (IG-028, Keyence Corp) mounted in front of the body restraint apparatus. The micrometers had a measurement range of 28 mm, a spatial resolution of 5 μm, and a temporal resolution of 490 μs. One bilateral homologous pair of whiskers was selected for monitoring. Selection was based on (1) the ability to fully capture the whisking motion on the micrometer array and (2) the ability to actuate the whisker on the paralyzed side with FES. A small polyimide tube (0.36 mm diameter) was placed on the selected pair of whiskers to facilitate its detection by the micrometer arrays (Heaton et al., 2008). The spatial sensitivity settings of the micrometers were adjusted such that they only detected the whisker in the tube and not the other whiskers.

### Data acquisition and FES

The laser micrometer output and EMG signals from the implanted microwires were simultaneously recorded at 3052 samples/s (PZ2 preamplifer, RZ2 processor, Tucker-Davis Technologies). EMG signals were recorded differentially with respect to the subcutaneous reference wire. Pairs of digitized EMG channels were then subtracted to yield the bipolar EMG signal used in offline analyses and online stimulus triggering. Electrical stimulation delivered to the microwires consisted of trains of charge-balanced, biphasic square pulses (IZ2H stimulator, Tucker-Davis Technologies). Pulse amplitude, pulse width, number of pulses, and pulse frequency were the adjustable stimulation parameters used to achieve different whisker motions. For closed-loop, EMG-triggered FES, the digital signal processors of the RZ2 were programmed for real-time EMG signal conditioning (bipolar subtraction, rectification, and 10–1,000 Hz bandpass filtering). Stimuli were then triggered when the conditioned signal amplitude reached an experimentally defined threshold, determined by trial and error at the beginning of the closed-loop sessions. A trigger lockout period starting with the first pulse and ending 20 ms after the end of the pulse train was imposed to prevent stimulus artifacts from triggering the stimulator.

### Data analysis

In offline analyses, the EMG envelope was estimated by bandpass filtering (5–100 Hz passband), rectifying, and then lowpass filtering (10 Hz cutoff) the raw bipolar signal. Power spectra of both the whisking signal and EMG envelope were computed using Welch's averaging method on 1-s, Hamming-windowed data segments with 50% overlap. Time delays between local peaks in the EMG envelope and whisking were used to assess the lag between these signals. Pearson's correlation coefficient was used to assess the linear relationship between peak whisking and EMG envelope amplitudes. For comparison of FES effects across animals, we identified the stimulus intensity required to evoke a 2 ± 0.2 mm whisker protraction, where protraction amplitude was defined as the peak positive displacement after subtracting the baseline mean. Closed-loop stimulation effects were summarized by stimulus-triggered average displacement of both paralyzed and intact whiskers, with 95% confidence intervals based on the t-distribution. The onset and duration of the post-stimulus protraction were defined by the first and last occurrences of positive displacement (i.e., lower confidence bound >0) in these averages, averaged across animals.

## Results

We developed a rodent model for studying FES-based facial reanimation. Optical micrometers (Figure 1A) were used to track a pair of whiskers bilaterally (Figure 1B) in head-fixed rats (Figure 1C). The facial musculature of the rats was implanted with microwires to allow both EMG recording and FES of facial muscles (Figure 1D). This system allowed us to study, at high spatiotemporal resolution, facial movements resulting from either volitional or artificial (i.e., FES) commands.

### Whisking and EMG activity

**Figure 2:**
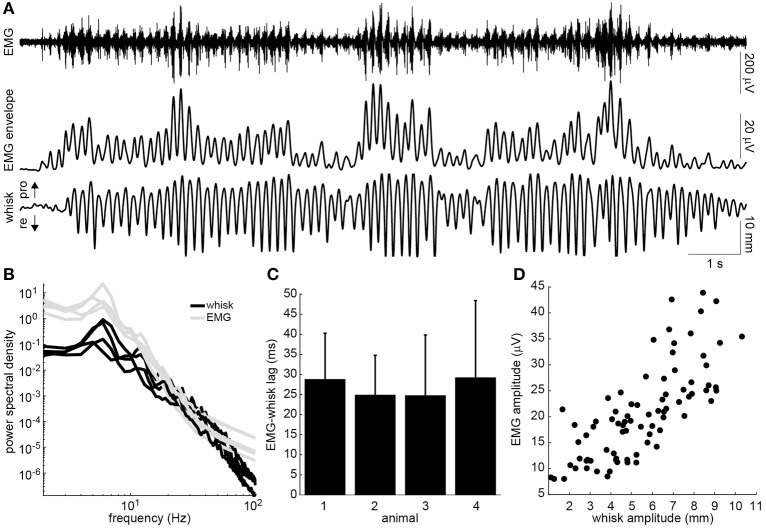
**Relationship of intrinsic muscle EMG to ipsilateral whisking. (A)** Example raw EMG recorded from the intrinsic muscle (top), envelope of the EMG signal (middle), and whisker motion (bottom). Directions of whisker protraction (pro) and retraction (re) are indicated. **(B)** Power spectra of the measured whisker (black) and EMG envelope (gray) signals for four rats. Note the prominent peak around 6 Hz. **(C)** Lag between the peaks of the EMG envelope and the corresponding protraction peaks of the whisker displacement (mean + standard deviation). **(D)** Relationship between the peak protraction amplitude and the peak EMG amplitude for the data shown in A (*r* = 0.75).

The head-fixed rats engaged in bouts of volitional, rhythmic whisking behavior (Figure 2A). This behavior could be produced spontaneously but often needed prompting by delivering scents near the nose. Rhythmic activity in the EMG of the whisker-protracting muscles corresponded with rhythmic motion of the tracked whisker on the intact side of the face (Figure 2A). To quantify the EMG-whisking relationship, the envelope of the EMG signal was computed using a zero-phase low pass filter. The dominant frequency of whisking and of the EMG envelope oscillation was 6 Hz in all four rats (Figure 2B). The EMG envelope led the whisker motion by an average of 28.7, 24.9, 24.7, and 29.2 ms in each of these animals, respectively (Figure 2C). Thus, the timing of each of cycle of the whisker motion could be accurately determined from the EMG. In one case, amplitude of the whisker motion could also be inferred from the EMG. The best observed correlation between peak whisk amplitude and peak EMG envelope amplitude was 0.75 (Figure 2D). However, in the other animals the whisk amplitude estimate was less reliable, with whisk-EMG correlations of 0.35, 0.32, 0.24, likely due to suboptimal electrode placement.

**Figure 3:**
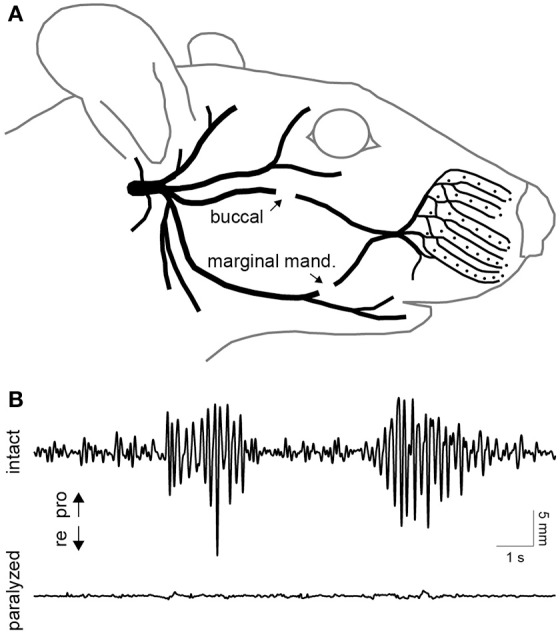
**Facial nerve transection and paralysis. (A)** Illustration of the peripheral branches of the facial nerve distal to the stylomastoid foramen. The two sites of transection are indicated by arrows. **(B)** Typical whisking behavior recorded simultaneously on the intact (top) and paralyzed (bottom) side. Whisker movements were recorded 10 days after transection of the buccal and marginal mandibular branches of the facial nerve on the paralyzed side.

A unilateral transection of the buccal and marginal mandibular branches of the facial nerve was performed in two of the four rats (Figure 3A). This procedure was effective in focally paralyzing the whisker pad. Rhythmic whisking was completely absent on the paralyzed side (Figure 3B). Only miscellaneous fibrillations up to 0.5 mm were present in the de-efferented whiskers. For over a month after the lesion, the rats maintained proper weight and eating habits and exhibited no self-injurious behavior.

### FES of intrinsic muscles

**Figure 4:**
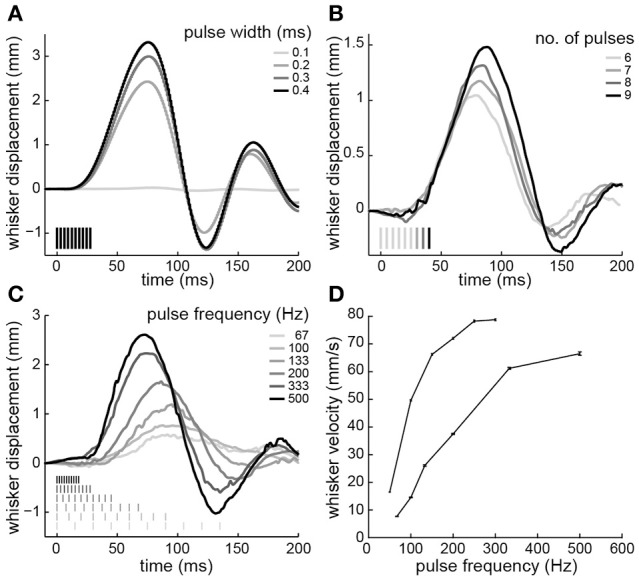
**Whisker motion resulting from electrical stimulation of paralyzed intrinsic muscles. (A)** Displacements produced by varying pulse widths (10 pulses at 333-Hz; animal 3). **(B)** Varying number of pulses (200-Hz frequency, 0.3-ms width; animal 4). **(C)** Varying pulse frequency (10 pulses, 0.3-ms width; animal 4). **(D)** Whisker velocity as a function of pulse frequency (10 pulses, 0.3-ms width; animals 3 and 4). Error bars are 95% confidence intervals on the slope of the initial protraction. Pulse amplitudes were 45 μA in animal 3 and 85 μA in animal 4. In **(A–C)**, time 0 is the start of the stimulus train. Stimulus pulses are indicated by vertical lines. Positive and negative displacements correspond to whisker protraction and retraction, respectively.

Next, electrical stimuli were delivered to the paralyzed intrinsic muscles to reanimate the whiskers. Trains of current-controlled square pulses were delivered between the pair of electrodes that produced whisker motion at lowest current amplitude. Across four testing sessions, the current to produce a 2-mm protraction was 43 ± 6 μA (animal 3) and 98 ± 13 μA (animal 4), when using 0.3-ms pulse widths, 10 pulses, and 200-Hz pulse frequencies. We characterized how variations in these stimulus parameters changed the evoked whisker motion. Increases in whisker displacement could be produced by increasing pulse width (Figure 4A), number of pulses (Figure 4B), or pulse frequency (Figure 4C). However, only pulse width affected amplitude without also changing the shape or timing of the motion. Increasing the number of pulses increased the amplitude and duration of the motion (Figure 4B). For both animals the duration, measured as time to peak amplitude, increased linearly, with a mean slope of 2.7 ± 0.37 ms/pulse when stimulating at 200 Hz. Increasing pulse frequency increased the amplitude and the velocity of the protraction (Figure 4C). The relationship between pulse frequency and whisker velocity, measured as the slope of the initial protraction for each frequency, was steeper in one animal and more graded in the other (Figure 4D). The results demonstrate that the amplitude and shape of the whisker motion can be controlled by stimulus pulse trains.

### Contralaterally-triggered FES

**Figure 5:**
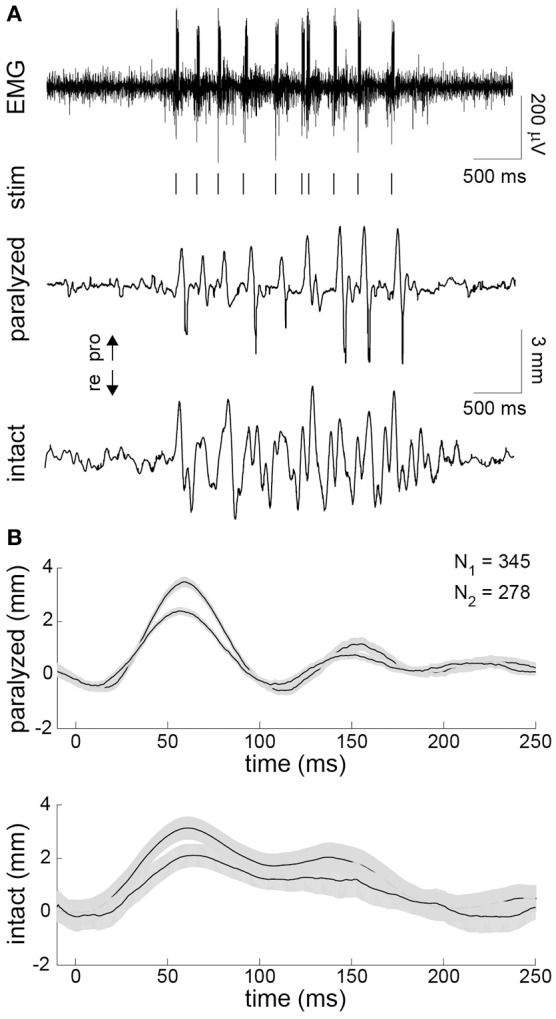
**Contralaterally-triggered electrical stimulation to restore symmetric whisking. (A)** Typical result during one burst of exploratory whisking. EMG was recorded on the intact side (top). The EMG was bandpass filtered and thresholded in real-time to produce stimulus triggers (vertical lines under EMG). The series of stimulus trains (10 pulses/train delivered at 200 Hz with 0.3-ms pulse width and 45-μA (animal 3) or 95-μA (animal 4) pulse amplitude) produced whisker motion on the paralyzed side (middle) that approximated the motion seen on the intact side (bottom). **(B)** Stimulus-triggered average of whisker displacements on the paralyzed (top) and intact (bottom) side for animals 3 and 4 compiled from all 345 and 278 stimulus trains delivered during the closed-loop experiment, respectively. Ninety-five percent confidence intervals on the mean are shown in gray.

Finally, in the unilaterally paralyzed rats, we used EMG activity recorded on the intact side to trigger stimulation of the paralyzed intrinsic muscles. The EMG was bandpass filtered and thresholded in real-time to generate stimulus triggers (Figure 5A). A train of stimulus pulses with fixed parameters was delivered immediately after each trigger. The stimulus parameters were chosen to achieve a whisker motion comparable to that observed on the intact side. A typical example of the EMG-triggered stimulation is shown in Figure 5A. The system was able to accurately deliver stimuli during each protraction of the intact whisker, as expected based on the preceding analyses. However, the overall symmetry between the intact and paralyzed whiskers was limited by several factors, including the inability to actively control retraction. To summarize the results, we computed the stimulus-triggered average motion of the paralyzed and intact whiskers in both animals (Figure 5B). The mean evoked motion on the paralyzed side had a peak amplitude and timing that was very similar to the mean intact-side protraction. Average onset of the post-stimulus protraction was 27 ms (paralyzed) and 24 ms (intact). The duration of post-stimulus motion was 150 ms (paralyzed) and 156 ms (intact). The average whisker motion of both sides was triphasic, although the intact motion was more variable being drive volitionally (Figure 5B).

## Discussion

In this study, we developed and tested a rodent model of closed-loop FES-based facial reanimation in the setting of unilateral facial paralysis. A primary strength of the model was the ability to track the relevant facial feature (i.e., whiskers) with high spatiotemporal resolution. The documented whisking behavior confirmed earlier findings. Prior studies have observed 5–6 Hz whisking frequencies in head-fixed rats (Gao et al., 2001). This is in contrast to the significantly higher, 6–12 Hz whisking frequencies seen in unrestrained rats (Hill et al., 2008). Also, the observed 24–29 ms lag between intrinsic muscle EMG and whisker protraction is nearly equivalent to a prior report (Berg and Kleinfeld, 2003). This lag can be attributed to the viscoelastic properties of the whisker pad. Electrical stimulation of the intrinsic muscles has previously been shown to protract the whiskers (Hill et al., 2008). However, here for the first time we documented how protraction varied with stimulation parameters. The velocity, peak amplitude, and duration of the protraction could largely be controlled through the number of stimulus pulses and pulse frequency. Finally, we demonstrated a closed-loop, contralaterally-triggered FES strategy that has been proposed for dynamic facial reanimation in humans (Cao et al., 2008; Griffin and Kim, 2011).

A limitation of the animal model is the difficult in durable placement of electrodes in the intrinsic muscles. These small, sling-like muscles around the base of each whisker cannot be directly visualized during implantation. As a result the electrode placement, and thus the recording and stimulation effects, can vary. This likely explains variability in the results, include the relationship between EMG amplitude and protraction amplitude and the relationship between whisker velocity and stimulus pulse frequency. A second limitation, which impacted the ability to restore symmetric whisking, is not actively controlling retraction. Whisker retraction is controlled by the nasolabialis and maxillolabialis muscles (Berg and Kleinfeld, 2003). However, these muscles pull the whiskers out of the horizontal plane along dorsal-posterior and ventral-posterior trajectories, respectively (Hill et al., 2008). The vertical components of these motions could not be captured by our micrometers, but could be monitored if the setup was extended to including two orthogonal micrometers on each side of the face (Hill et al., 2008). Alternatively, simultaneous stimulation of both retracting muscles can produce a mostly in-plane, posterior movement (Hill et al., 2008). Initial experiments found it quite difficult to get the concurrent electrode placement in all four muscles bilaterally. Thus, we relied on the passive retraction that occurs following active protraction (Berg and Kleinfeld, 2003).

Another potential limitation involves the FES strategy itself. Most etiologies of unilateral facial paralysis leave facial sensation intact, as the trigeminal nerve is not affected. Therefore, FES has the potential to activate sensory axons associated with nociceptors, evoking painful percepts. However, several lines of evidence suggest this issue does not completely undermine the strategy. No signs of pain (flinching, blinking, or vocalizing) were observed in our experiments. A prior study observed that rabbits initially flinched in response to orbicularis oculi stimulation, but signs of pain diminished over time (Otto, 1997). There is evidence that FES paradigms including interferential stimulation (McDonnall et al., 2009) and low-intensity multichannel stimulation (Somia et al., 2001) render facial muscle stimulation functional but not painful. Finally, facial FES has been achieved in humans reporting only mild pain (McDonnall et al., 2009; Frigerio et al., 2015).

An issue that was not studied, but should be considered, in this model of chronic facial paralysis is the time-dependent effects of denervation and the modulation of these effects by FES. Denervation is associated with muscle atrophy, which in the rat can result in a 50% loss of muscle weight after 2 weeks (Ohira, 1989). Importantly for FES, denervation also causes a transient increase in excitability, due in part to increased sensitivity to acetylcholine, followed after a few days by decreased excitability due to Wallerian degeneration of the axons distal to the injury (Sunderland, 1978). Nevertheless, prior animal studies of facial FES have elicited functional movement (e.g., complete eyelid closure) for several months after paralysis (Salerno et al., 1991; Otto, 1997; Sachs et al., 2007). This may be explained in part by demonstrations that FES can prevent and even reverse the effects of denervation, both in facial muscles (Salerno et al., 1991) and non-facial muscles (Eberstein and Eberstein, 1996). Reinnervation, potentially from motor axons in surrounding, non-denervated muscles, may also play a role (Sachs et al., 2007). Our rodent model of facial paralysis provides another means to explicitly study the interaction of muscle denervation and FES, with the benefit of precise quantification of muscle activation through whisker monitoring.

Previous research on contralaterally-triggered facial FES has been done in larger animal models and humans over the course of several decades (Zealear and Dedo, 1977; Tobey and Sutton, 1978; Broniatowski et al., 1987, 1989, 1991; Cao et al., 2009; McDonnall et al., 2009; Kurita et al., 2010; Frigerio and Cavallari, 2012). However, the therapy has not advanced beyond proof of concept. Our motivation for developing a less expensive, more quantifiable animal model for this therapy was to move toward clinical translation by improving performance through advanced closed-loop controllers. In the present study, as in all previous work, the closed-loop controller was simple: deliver hand-tuned, fixed-parameter stimuli to the paralyzed muscle when triggered by thresholded EMG activity of the homologous intact muscle. Myriad strategies, including iterative learning control (Bristow et al., 2006), adaptive feedforward control (Abbas and Triolo, 1997), and supervised learning with a distal teacher (Jordan and Rumelhart, 1992), could be used to automatically tune a mapping between EMG activity and stimulation parameters based on measured facial asymmetries to yield superior performance. The animal model developed here provides an improved platform with which to test these advanced controllers. We believe this is a necessary step to develop a therapy that could improve the quality of life of thousands of patients with facial paralysis.

## Ethics statement

This study was carried out in accordance with the recommendations of the National Institutes of Health guidelines on the use of animals. The protocol was approved by the University of Pennsylvania Institutional Animal Care and Use Committee.

## Author contributions

AR and TL designed the study. MA, Jd, and AR conducted the experiments and analyzed the results. All authors contributed to writing the manuscript.

### Conflict of interest statement

The authors declare that the research was conducted in the absence of any commercial or financial relationships that could be construed as a potential conflict of interest.
